# Safety, tolerability and pharmacokinetics of subcutaneous cefazolin as an alternative to intravenous administration

**DOI:** 10.1093/jac/dkae397

**Published:** 2024-12-13

**Authors:** Fionnuala Murray, Okhee Yoo, Samuel Brophy-Williams, Matthew Rawlins, Steven C Wallis, Jason A Roberts, Edward Raby, Sam Salman, Laurens Manning

**Affiliations:** Department of Infectious Diseases, Fiona Stanley Fremantle Hospitals Group, Murdoch, Western Australia 6150, Australia; Wesfarmers Centre of Vaccines and Infectious Diseases, Telethon Kids Institute, University of Western Australia, Perth, Western Australia 6009, Australia; Pharmacy, School of Allied Health, The University of Western Australia, Perth, Western Australia 6009, Australia; Institute for Paediatric Perioperative Excellence, The University of Western Australia, Perth, Western Australia 6009, Australia; Department of Infectious Diseases, Fiona Stanley Fremantle Hospitals Group, Murdoch, Western Australia 6150, Australia; Department of Pharmacy, Fiona Stanley Fremantle Hospitals Group, Murdoch, Western Australia 6150, Australia; Department of Immunology, PathWest Laboratory Medicine, Fiona Stanley Hospital, Murdoch, Western Australia 6150, Australia; University of Queensland Centre for Clinical Research, Faculty of Medicine The University of Queensland, Brisbane, Australia; University of Queensland Centre for Clinical Research, Faculty of Medicine The University of Queensland, Brisbane, Australia; Herston Infectious Diseases Institute (HeIDI), Metro North Health, Brisbane, Australia; Departments of Pharmacy and Intensive Care Medicine, Royal Brisbane and Women’s Hospital, Brisbane, Australia; Division of Anaesthesiology Critical Care Emergency and Pain Medicine, Nîmes University Hospital, University of Montpellier, Nîmes, France; Department of Infectious Diseases, Fiona Stanley Fremantle Hospitals Group, Murdoch, Western Australia 6150, Australia; Department of Microbiology, PathWest Laboratory Medicine, Fiona Stanley Hospital, Murdoch, Western Australia 6150, Australia; Wesfarmers Centre of Vaccines and Infectious Diseases, Telethon Kids Institute, University of Western Australia, Perth, Western Australia 6009, Australia; Department of Infectious Diseases, Fiona Stanley Fremantle Hospitals Group, Murdoch, Western Australia 6150, Australia; Wesfarmers Centre of Vaccines and Infectious Diseases, Telethon Kids Institute, University of Western Australia, Perth, Western Australia 6009, Australia; Medical School, Faculty of Health and Medical Sciences, The University of Western Australia, Harry Perkins Research Institute, Fiona Stanley Hospital, PO Box 404, Bull Creek 6149, Crawley, Western Australia 6009, Australia

## Abstract

**Background:**

Subcutaneous (SC) administration of antibiotics is a practical alternative to IV administration. Cefazolin is widely used for skin and soft tissue infections and other complex infections by IV administration.

**Methods:**

In this prospective, cross-over self-controlled study, a single dose of SC cefazolin was administered to 15 stable inpatients established on IV cefazolin as part of their management plan. The equivalent dose of cefazolin was diluted in 50 mL of normal saline via gravity feed over 30 min. Venous blood samples were collected at baseline and 0.5, 1, 2, 4 and 8 h following both the SC and IV doses. Antibiotic concentrations were measured using UPLC-MS/MS. Pharmacokinetic data were analysed using a non-linear mixed-effects modelling approach. Pain scores and infusion site reactions (oedema/erythema) were evaluated.

**Results:**

SC cefazolin was well tolerated. The bioavailability of SC administration was 74.8% (95% CI 66.7%–81.7%). Slower absorption from SC tissue was associated with a BMI of ≥30. Lower peak, and higher trough concentrations were observed with SC administration. Although lower bioavailability was observed with SC administration, the PTA for unbound drug concentrations greater than the MIC for more than 90% of the time between doses was higher for SC compared with IV administration at MICs between 0.25 and 4 mg/L. Simulated SC doses of 3 g twice daily had similar PTA to standard IV dosing of 2 g three times daily. A simulated 6 g continuous 24 h infusion of SC cefazolin had a favourable pharmacokinetic profile.

**Conclusion:**

SC cefazolin appears to be well tolerated, with an improved pharmacokinetic profile compared with IV administration. Either 3 g twice daily, or 6 g as a 24 h SC infusion could be considered for future evaluation.

## Introduction

Cefazolin is a first-generation parenteral cephalosporin antibiotic with activity against methicillin-susceptible stains of *Staphylococcus aureus* and CoNS, as well as *Streptococcus* species and some Gram-negative species including *Escherichia coli*, *Proteus mirabilis*, *Klebsiella* spp. and *Moraxella* spp.^1^ It is administered IV to treat skin and soft tissue infections, as well as more deep-seated infections such as endocarditis and bone and joint infections with susceptible organisms. It is the mainstay of surgical prophylaxis.

Subcutaneous (SC) administration has many advantages over alternative methods of parenteral antibiotic administration such as IV or intramuscular (IM), including ease of administration without the need to secure sustained vascular access. This in turn reduces the risk of associated complications including extravasation of medication, thrombophlebitis, secondary bloodstream infection, and missed antibiotic doses during inpatient admissions due to inability to secure IV access. Another important consideration is the fact that patient satisfaction and consumer experience is significantly impacted by patient discomfort from multiple attempts to secure IV access.^2^ In cases where prolonged parenteral antibiotic therapy is required, a long-term venous access device is necessary, which often requires specialist teams for insertion, thereby creating a further barrier to timely discharge, resulting in prolonged hospital admissions and increased healthcare costs. Problems that arise in the community such as blockage, thrombosis and secondary infection, which can result in readmission, also have significant cost implications for the healthcare system. The use of SC administration of antibiotics also offers an important vein preservation strategy in patients with chronic kidney disease progressing towards renal replacement therapy.^3^ Finally, although IM administration is approved for numerous antibiotics and can be used in certain instances to avoid the need for IV access, it is associated with significant patient discomfort and hence is often not utilized.^4^

SC administration of antibiotics was first described over 50 years ago and has been used for many years across Europe, particularly in France.^5,6^ Prior studies have reported good safety and tolerability, as well as favourable pharmacokinetic (PK) profiles associated with SC administration of a number of other time-dependent antibiotics including ceftriaxone, ertapenem and teicoplanin.^7–10^

To date, there are no human data describing the administration of cefazolin via the SC route, although favourable PK profiles and safety have been described in a porcine model.^11^ We hypothesized that SC administration of cefazolin would be safe, well tolerated and have favourable PK/pharmacodynamic (PK/PD) parameters when compared with IV administration.

## Methods

### Study design

We conducted a prospective, cross-over self-controlled trial, with PK data collected for both SC and IV administration for each participant. The primary objective was to describe the PK of SC administration of cefazolin compared with IV. The secondary objectives were to assess the safety and tolerability of SC administration of cefazolin. This study was approved by the South Metropolitan Health Service Ethics Committee (RGS5105) and prospectively registered (Australia and New Zealand Clinical Trials Registry; ACTRN12623000577617). All participants provided written informed consent.

### Participants

Eligible participants recruited into the study were aged 18 years or older, admitted to Fiona Stanley Hospital, Western Australia and established on cefazolin as part of their treatment plan. Participants had to be clinically stable at the time of enrolment, defined as not requiring ICU/high-dependency unit admission or having had a medical emergency team (MET) call within the preceding 24 h. Full exclusion criteria are provided in Table S1, available as Supplementary data at *JAC* Online. Sample size calculation for detection of 10% lower bioavailability of SC dosing compared with IV dosing and to demonstrate equivalent bioavailability between the two routes of administration, based on FDA/EMA guidance for bioequivalence, would require 15 participants with complete datasets.^12^

### Study interventions

After at least one dose of IV cefazolin, plasma samples were collected from stable participants following an IV dose of cefazolin to establish individual PK parameters. Participants then received a single SC infusion of cefazolin matching the dose (1 or 2 g) and dosing interval (6-, 8- or 12-hourly) prescribed by the treating team. The prescribed dose was diluted in 50 mL of normal saline delivered into the SC tissues of the lower anterior abdominal wall via gravity feed over approximately 30 min, using a 22-gauge SC catheter (BD Saf-T-Intima, BD Medical, Mississauga, ON, Canada). A saline flush (10–20 mL) was given at the end of the infusion via gravity feed over 5–10 min.

### PK samples

For both IV and SC dosing, venous blood (3 mL) was collected into a lithium heparin tube immediately prior to dosing, and 30 min and 1, 2, 4 and 8 h post commencement of the dose. A sample was collected at 6 h instead of the 8 h sample in participants receiving 6-hourly dosing. Additional venous blood samples were collected at 12 h for participants on twice-daily dosing. Baseline haematocrit, serum creatinine (Cr) and albumin measures were obtained from blood tests collected as part of routine clinical care on the day of dosing. CL_CR_ was subsequently calculated using the Cockcroft–Gault equation.

Samples were immediately placed on ice and then centrifuged for 10 min at 2000 rpm within 1 h of collection. Plasma samples were stored at −80°C and transferred to the analytical laboratory on dry ice.

To isolate the free fraction of cefazolin, plasma samples underwent ultracentrifugation at 37°C with Centrifree devices (Merck Millipore, Tullagreen, Ireland) and the ultracentrifuged plasma was then processed as a typical plasma sample in order to obtain the unbound concentration. Total and unbound concentrations of cefazolin in plasma were measured using a validated UPLC-MS/MS method.^13^ The lower limit of quantification for total and unbound concentrations was 1.0 mg/L. Test samples were assayed in batches alongside calibrators (1–500 mg/mL) and quality controls and results subjected to batch acceptance criteria (US FDA, Guidance for Industry: Bioanalytical Method Validation, May 2018). Precision and accuracy were <5%.

### Safety and tolerability

Tolerability of SC administration was assessed at the same timepoints as plasma sampling using the numerical rating scale (NRS) for pain with scores from 0 to 10, with 0 representing ‘no pain’ and 10 representing the ‘worst pain imaginable’.^14^ Skin at the SC infusion site was evaluated for local erythema and oedema. Erythema was scored on a 0–4-point scale with 0 representing ‘no erythema’ and 4 representing ‘severe erythema to slight eschar formation.’ Oedema was scored using a 0–4-point scale with 0 representing ‘no oedema’ and 4 representing ‘severe oedema (raised more than 1 mm and beyond exposure area)’.

### Population PK modelling

Unbound cefazolin concentrations were utilized to construct the population PK model using NONMEM (v 7.5.1, ICON Development Solutions, Ellicott City, MD, USA) with a GFortran 4.6.0 compiler, supported by Perl-Speaks-NONMEM (PsN) and Pirana.^15^ Parameter estimation was conducted via the first-order conditional estimation method with interaction. Data management was facilitated using R version 4.1.3 (R Foundation for Statistical Computing, Vienna, Austria), Microsoft Excel and Power BI Desktop (Version: 2.126.1261.0). Model diagnostics and graphical evaluation of the results were supported by the Xpose4 package.

Statistical significance for adding extra parameters to nested models was evaluated using the objective function value (OFV). A decrease in OFV (ΔOFV) of 3.84 between two nested models that differed by a single parameter was deemed statistically significant at the 5% level (*P* < 0.05). Furthermore, the selection of the model was informed by graphical analyses of goodness-of-fit plots, simulation-based prediction-corrected visual predictive checks (VPCs), and credible parameter estimates with satisfactory precision.

**Figure 1.**
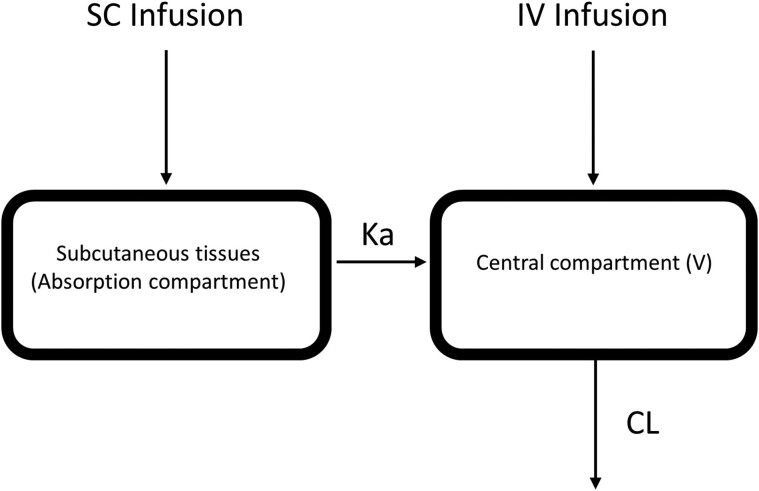
Schematic representation of the one-compartment PK model for unbound (free) plasma cefazolin concentrations.

The dose of 2 g of cefazolin sodium was converted to 1.908 g of cefazolin base for the analysis. SC infusion was modelled as input into the depot compartment, with bioavailability and absorption rates to the central compartment (Figure 1). The data were analysed using both one- and two-compartment models that incorporate first-order elimination processes. Various absorption models, including zero-order, first-order and transit models, were tested. Interindividual variabilities (IIVs) of population PK parameters were estimated, and proportional residual error was evaluated, assuming a normal distribution with a mean of zero.

Initially, allometric scaling was tested to evaluate the influence of body weight (WT) on clearance (CL) and volume of distribution (*V*), applying exponents of 0.75 and 1, respectively. Once a satisfactory structural model was established, the final PK model for unbound cefazolin was developed by assessing the impact of covariates. We applied a forward inclusion method (*P* < 0.05; ΔOFV > 3.84; 1 df) followed by a backward elimination process (*P* < 0.01; ΔOFV > 6.63; 1 df). The relationships of Cr and CL_CR_ on CL, and BMI on relative bioavailability (F1) and absorption rate (Ka) were tested for covariate relationships.

Using the final model, alternative dosing regimens and modes of administration were simulated to evaluate steady-state conditions in relation to the PTA. For the simulations, steady-state concentrations were utilized 24 h after the initial dosing. Steady state was assumed to occur 8 h post dosing, based on the estimated *t*_½_ of 1.7 h from our model parameters. In addition to the standard dosing of 2 g three times daily for IV and SC dosing, additional dosing SC regimens of 3 g twice daily and 6 g given as a continuous infusion over 24 h were simulated. For each, a population of 1000 individuals was simulated for each regimen. Covariates were uniformly distributed across the simulated population, with proportions based on the demographics of the study population. For the purposes of the current study, we assumed that target attainment was unbound concentrations greater than the MIC for 90% of the time between injections. The PTA was calculated as the proportion of patients attaining this target according to the different route of administration and MIC.

## Results

### Baseline characteristics

A total of 27 participants were screened, with 15 eligible participants recruited. Fourteen participants were being treated for diabetes-related foot infections and one for infective endocarditis due to *S. aureus*. All were male. The median (IQR) age was 62 (51–75) years. The median (IQR) weight was 96 (90–115) kg, with a median BMI of 29.7 (27.1–35.6) kg/m^2^. Six participants had a BMI of ≥30 kg/m^2^. The median (IQR) Cr and CL_CR_ were 75 (58–122) μmol/L and 98 (66–153) mL/min, respectively. Most (12; 80%) were receiving 2 g three times daily, whilst twice-daily (2: 13%) and four-times-daily (1; 7%) regimens were less frequent. All participants completed the study. Two participants did not receive the full dose of SC cefazolin as planned. One had the infusion disconnected in error prior to completion, having received 1.4 g, and another received 1.8 g, as the last 6 mL failed to progress through the gravity feed tubing due to a kink at the SC catheter insertion site.

### Tolerability and safety

Reported NRS pain scores related to SC infusion ranged from 0 to 5. Five participants reported a pain score of 0 throughout the SC infusion and the subsequent 24 h. Nine participants reported mild pain (NRS 1–3) directly associated with the SC infusion of the antibiotic. Moderate pain (NRS 5) was reported by one participant. No pain or discomfort was reported by participants after completion of the SC infusion.

Seven participants had mild SC infusion site swelling corresponding to a score of 1. No participants had moderate or severe SC infusion site oedema. All participants who experienced mild infusion site oedema had complete resolution of this by 2 h post infusion. There was no association between infusion site oedema and reported NRS pain scores.

No infusion site erythema was observed in any participant during or after the SC infusion. There were no serious adverse events (grade 2 or higher) reported during the study.

### PK modelling

One hundred and seventy-five plasma samples were collected. For the population PK analysis, 167 unbound cefazolin concentrations were included. Six sample concentrations were below the limit of quantification (1 mg/L) and three samples had insufficient volume to allow analysis; these samples were excluded from the analysis. The observed serum concentration–time profiles were best described by a one-compartment model featuring first-order input and first-order elimination. The model-derived PK parameters included total CL, central *V* (*V*_c_), relative bioavailability from SC administration (F1), and the SC absorption rate constant (Ka). IIV was estimated for all PK parameters as shown. Allometric scaling of CL by 0.75 and *V* by 1 using weight was added *a priori*. However, incorporating a linear relationship between CL_CR_ and CL resulted in an overestimation of the WT term. This occurred because CL_CR_ was estimated using Cockcroft–Gault equation, which incorporates WT in its formula. This formula for estimating CL_CR_ is given by (140 − age in years) × (WT in kg) × CF)/(Cr in μmol/L), where CF (correction factor) is 1.04 for female participants and 1.23 for male participants.

**Figure 2.**
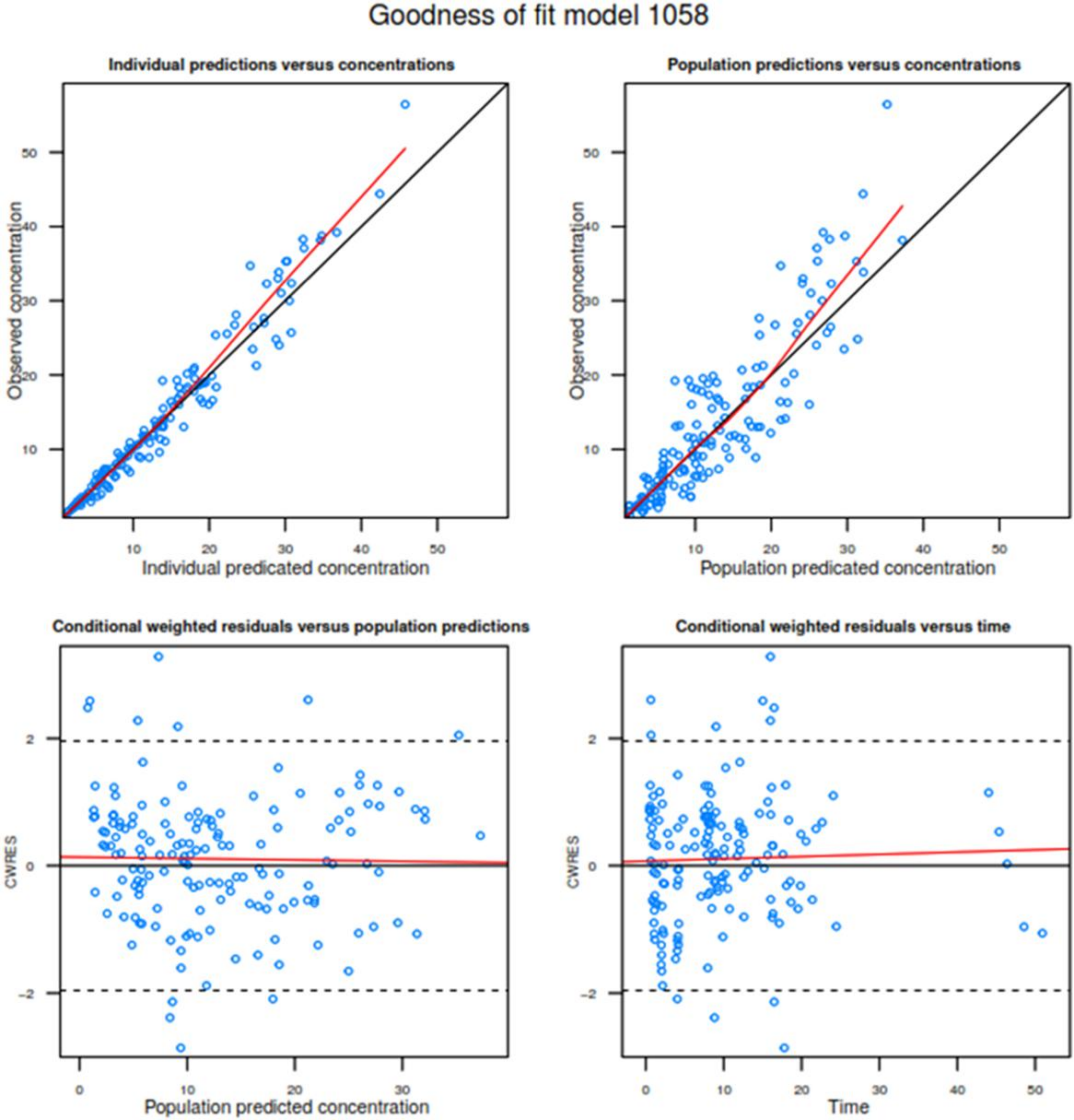
Goodness-of-fit (GOF) plots for the IV and SC cefazolin infusion final model. (a) Individual-predicted concentration versus observed concentration; (b) population-predicted concentration versus observed concentration; (c) conditional weighted residuals (CWRES) versus population-predicted concentration; (d) CWRES versus time. The red and black lines in (a) and (b) show regression lines, and the line of identity, respectively. The red and black lines in (c) and (d) show the zero reference line and a smooth non-parametric regression line, respectively.

**Figure 3.**
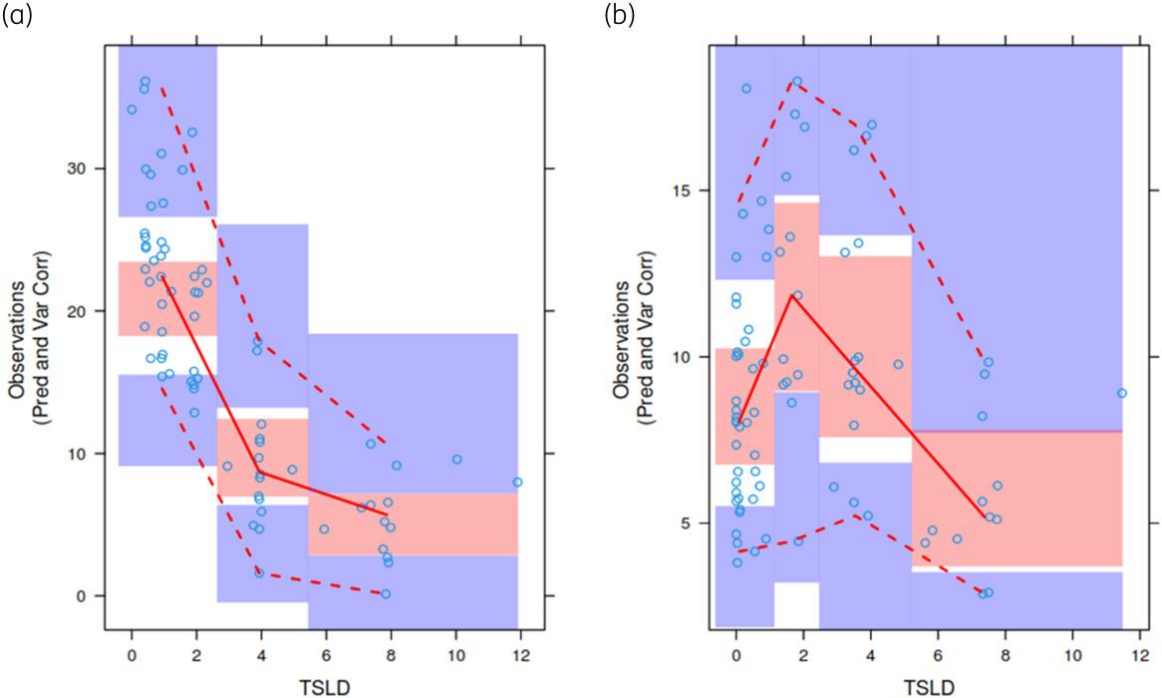
VPCs for unbound cefazolin concentrations (mg/L) in plasma after IV and SC infusions. (a) Dosing via IV infusion; (b) dosing via SC infusion. Open circles represent the measured concentrations of unbound cefazolin. Solid lines depict the median values of the observed data. Dashed lines indicate the 5th and 95th percentiles of the observed data. Shaded areas illustrate the 95% CIs for the simulated values from the PK model, with upper and lower shaded areas corresponding to the 95th and 5th percentiles, respectively, and the middle shaded area representing the 50th percentile. TSLD, time since last dose.

The addition of CL_CR_ as a covariate to CL improved the model. CL (L/h) = 19.6 × (CL_CR_/100), where 19.6 represents the typical population estimate. The inclusion of BMI as a dichotomous variable (with 30 kg/m^2^ as a cut-off) to Ka further improved the model. A BMI of ≥30 kg/m^2^ increased absorption time (^1^/_Ka_) by 2.5 times from the SC tissue compared with participants with a BMI of <30 kg/m^2^. The median (95% CI) bioavailability was 74.8% (66.7%–81.7%). The model-estimated population PK parameters for unbound cefazolin with their associated interindividual and residual variabilities and bootstrap results demonstrated good model fit (Table 1). Goodness-of-fit plots of the final population PK model and VPCs with the 90% prediction intervals are shown (Figures 2 and 3, respectively).

**Table 1.** Final population PK model parameters and bootstrap results for unbound (free) cefazolin

Parameter	Mean	%RSE	Bootstrap, median (95%CI)
OFV	400.015		398.3 (330.1–449.4)
Structural model parameters			
CL_CLCR_ (L·h^−1^)	19.6	8	19.6 (17.0–22.7)
*V*_c_, (L) 70 kg^−1^	48.7	7	48.6 (43.1–55.0)
F_SC_	0.744	7	0.742 (0.667–0.817)
Ka_SC_ (h^−1^)	0.759	18	0.782 (0.628–1.105)
Ka_SC,BMI_ (%)	59.8	16	61.9 (50.4 –79.2)
IIV (shrinkage, %)			
CL_CLCR_	29.1 (3)	15	28.2 (21.2–34.8)
*V*_c_	21.2 (12)	20	20.4 (11.3–28.0)
F_SC_	16.4 (24)	50	17.4 (3.2–31.8)
Ka_SC_	34.2 (19)	37	35.1 (19.2–53.3)
Residual errors			
Prop (%)	15.8 (15)	1	15.7 (13.1–17.9)

CL_CLCR_, total CL with effect of CL_CR_; F_SC_, absolute SC bioavailability; Ka_SC_, SC absorption rate constant; Ka_SC,BMI_, effect of BMI on Ka_SC_; prop, proportional error; %RSE, relative standard error [%RSE = 100 × (standard error/parameter estimate)]; IIV and residual error are presented as 100% $\times \sqrt{variability\;}estimate$.

**Figure 4.**
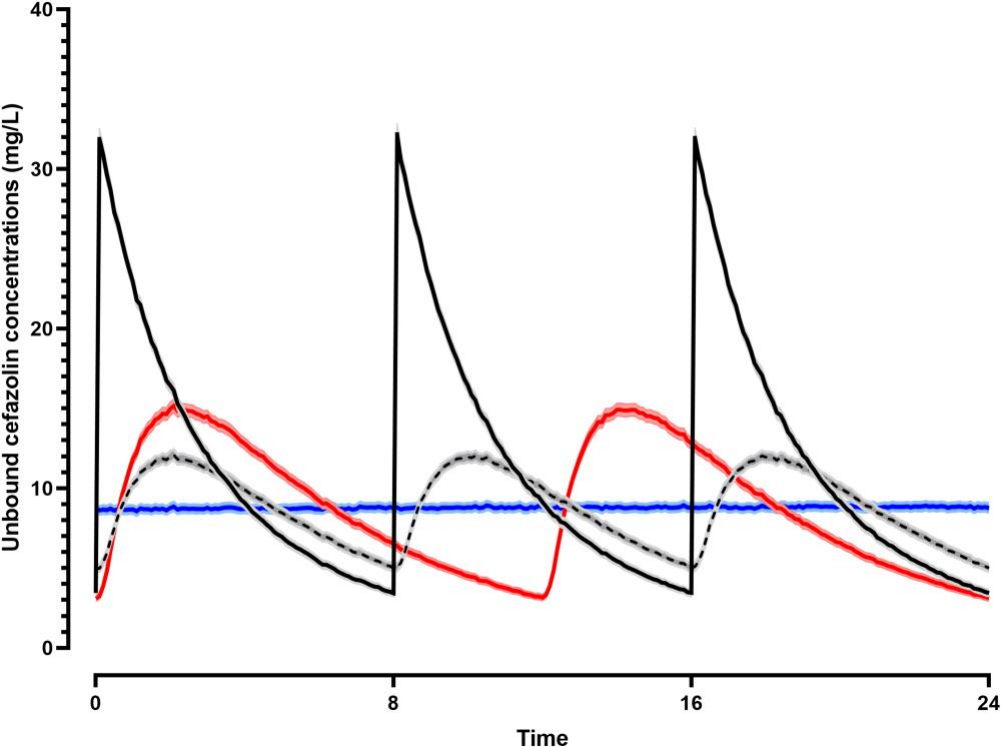
Simulated mean (with shaded simulated 95% CIs) steady-state unbound concentrations of cefazolin over 24 h. Standard IV dosing (2 g three times daily, black line) is compared with the same dose given SC (dashed). Simulated regimens of 3 g twice daily (red), and 6 g given SC as a continuous infusion (blue) are also shown.

**Figure 5.**
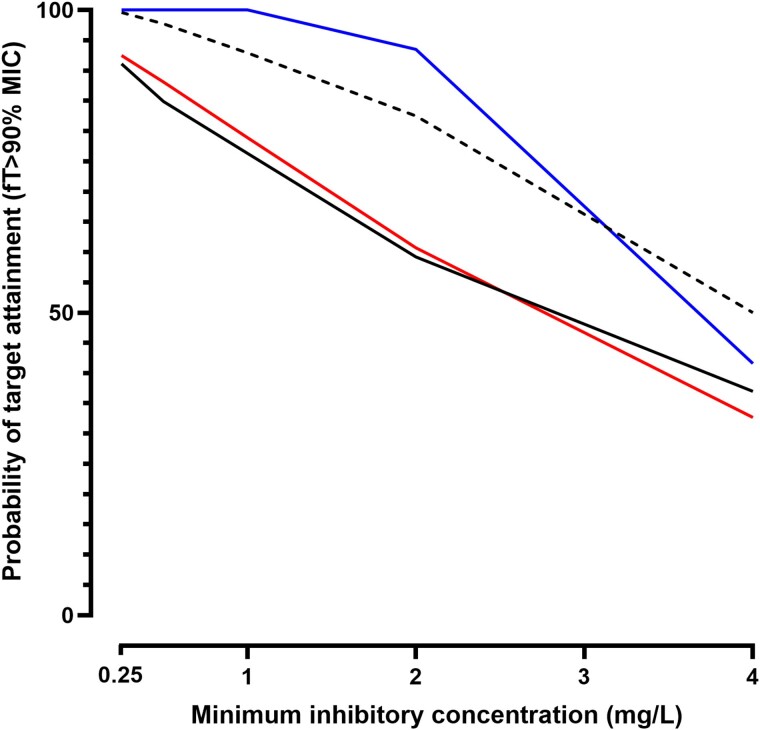
PTA, defined by unbound (free) cefazolin concentrations exceeding the MIC for 90% of the time between injections at steady state, according to possible MICs. Standard IV dosing (2 g three times daily, black line) is compared with the same dose given SC (dashed). Simulated regimens of 3 g twice daily (red), and 6 g given SC as a continuous infusion (blue) are also shown.

Using the final model, simulations demonstrated that cefazolin delivered SC had lower peak and higher trough concentrations, compared with IV dosing (Figure 4). Furthermore, the PTA at MICs between 0.25 and 4 mg/L was higher following SC delivery. Doses of 3 g delivered SC twice daily had very similar PTA to 2 g three times daily delivered IV. By contrast, if 6 g could be delivered as a continuous infusion over 24 h, the PTA would be higher than 2 g three times daily SC at all MICs except an MIC of 4. Continuous infusion had superior PTA to standard IV dosing at all MICs (Figure 5).

## Discussion

SC administration of antibiotics offers an attractive alternative to other parenteral routes of administration, particularly in situations where IV administration is challenging. In this study, we established that delivery of SC cefazolin appears to be safe and well tolerated. The PK profiles for SC administration demonstrated lower peak and higher trough concentrations resulting in a higher PTA at all clinically relevant MIC cut-offs despite lower bioavailability. A BMI of ≥30 kg/m^2^ was associated with delayed absorption from the SC tissue, which would enhance the favourable time-dependent PK characteristics of this route of administration. Bioavailability was 0.744. Neither BMI nor WT affected bioavailability (Table 1).

The bioavailability of SC administration was ∼75%. This was an unexpected finding, given most studies report good bioavailability of 96%–100% for other SC antibiotics.^9,16–20^ A possible reason for this observation is that the intended dose was not fully administered, with some drug remaining in the infusion bag. This was observed in two participants who didn’t receive the full dose as planned. In clinical practice, the importance of ensuring the full dose is delivered by SC infusion should be emphasized. Nevertheless, despite the lower bioavailability, the PK profiles obtained were still favourable, relative to IV dosing.

SC administration is a useful alternative in several scenarios. First, in settings where parenteral medications are still required but IV access is difficult to obtain. This might be relevant to smaller hospitals where ultrasound guidance or senior expertise may not be available out of hours. Second, due to the lower peak and higher troughs, the drug exposure profiles observed with SC administration might be seen as an advantage when cefazolin is used as surgical prophylaxis.^11^

Third, patients on haemodialysis or with chronic renal failure where once-daily dosing is recommended and where vein preservation is an important consideration.^3^ CL within our PK model was directly related to renal function, which is consistent with previous population PK models.^13,21^ Dose adjustments according to renal function for SC dosing should follow the same recommendations as for IV, given that the route of administration does not impact CL. Thus, a single daily SC-administered dose, with the potential to be administered in ambulatory settings, represents an ideal option in this group.

Using the population PK model, we simulated several different dosing regimens, including increased doses at longer dosing intervals, which could be explored in future studies. As an example, 3 g of cefazolin via SC infusion at 12-hourly dosing intervals had a very similar PTA to IV 8-hourly dosing. This would offer a practical alternative both for inpatients, where dose frequency is likely to impact on nursing workloads, and in ambulatory care settings, where twice-daily dosing may make outpatient care feasible. Furthermore, a 6 g continuous SC infusion over 24 h could be feasible, if demonstrated to be tolerable. These simulations provide reassurance that the proposed new dosing regimens will not compromise antibiotic efficacy, though safety and tolerability for patients should be evaluated in future observational studies.

A significant limitation of our study was that all participants were male, most of whom were being treated for diabetes-related foot infections. This patient cohort tends to have a higher BMI, and renal impairment is common.^22^ The inclusion of the effects of BMI and renal function in the model enhances the generalizability of the PK findings to other non-critically ill hospital patients; however, these findings are not applicable to critically ill patients due to the significant physiological changes that occur in this patient population and the impact of this on PK/PD parameters. Further work is required to demonstrate the safety and tolerability in other groups, such as women and children. As we only observed a single dose, exploring the tolerability of multiple doses should also be explored further.

### Conclusions

Administration of cefazolin via the SC route appears to be well tolerated and demonstrates favourable PK parameters for clinical use. Dosing regimens of 3 g, given SC twice daily or 6 g as a 24 h SC infusion could be considered as possible regimens for future evaluation.

## Supplementary Material

dkae397_Supplementary_Data

## Supplementary data

Table S1 is available as Supplementary data at *JAC* Online.
