# Supplementary material for: Safety, tolerability and pharmacokinetics of subcutaneous cefazolin as an alternative to intravenous administration
Source: J Antimicrob Chemother. 2024 Dec 13;80(2):347–53. doi: 10.1093/jac/dkae397 (PMC11787891; doi:10.1093/jac/dkae397)
Supplement: dkae397_Supplementary_Data [file dkae397_supplementary_data.docx]

**Safety, tolerability and pharmacokinetics of subcutaneous cefazolin as an alternative to intravenous administration**

Supplementary Data

Table S1 – Exclusion Criteria

| **Exclusion Criteria** |
| --- |
| - Patients not clinically stable, as defined by being in ICU, having had a MET call in the 24 hours before screening for enrolment. - Children <18 years - Patients whose treating team predict will cease cefazolin within 48 hours. - Patients receiving >2g cefazolin - Patients also taking medications predicted to interfere with cefazolin pharmacokinetics (valproic acid, probenecid) - Patients unable to give informed consent themselves. |
